# Ambiguities in Powder Indexing: the Impact of a Quaternary Lattice Metric Singularity on the Characterization of Mawsonite and Chatkalite

**DOI:** 10.6028/jres.111.029

**Published:** 2006-10-01

**Authors:** Alan D. Mighell

**Affiliations:** National Institute of Standards and Technology, Gaithersburg, MD 20899-8520

**Keywords:** ambiguities in powder indexing, Chatkalite, derivative lattices, *d*-spacings, indexing programs, Mawsonite, quaternary lattice metric singularity, powder indexing, specialized lattices

## Abstract

A lattice metric singularity occurs when unit cells defining two (or more) lattices yield the identical set of unique calculated *d*-spacings. The minerals Mawsonite and Chatkalite are of especial interest as both are characterized by tetragonal unit cells that correspond to the second member of a quaternary lattice metric singularity. This singularity includes lattices that are Cubic *I*, Tetragonal *P*, Orthorhombic *F*, and Orthorhombic *P*. The Mawsonite and Chatkalite lattices are unique in that they are highly specialized. In each case: (1) the determinative *c*/*a* ratio is very near 1/√2, (2) the symmetrical scalars of the reduced form [***a* · *a* : *b* · *b* : *c* · *c*** = 1:2:2] have greater specialization than required for the given reduced form type, (3) the tetragonal lattice has derivative lattices of higher symmetry, and (4) the powder pattern is highly compressed. Mawsonite and Chatkalite serve as exemplar-type compounds. Their tetragonal structure has important implications in structure determination using powder diffraction data. First, any cubic *I* lattice — established solely on the basis of indexing procedures — may actually be tetragonal or orthorhombic. Second, in establishing the lattice of an unknown, results from powder data indexing require routine confirmation by other techniques (e.g., single crystal, optical, etc.).

## 1. Introduction

Today a wide variety of crystal structures are solved using x-ray or neutron powder diffraction data in conjunction with Rietveld refinement techniques. The sequence of steps in the structure determination — including data collection, structure determination and refinement — are often integrated and highly automated.

A critical step in the solution process is the determination of a unit cell that defines the lattice. This is commonly done via analogy with related compounds or with an indexing program such as DICVOL9l [1] or TREOR [2]. Unfortunately, the cell determination process is not entirely straightforward and is sometimes fraught with pitfalls. In certain cases, a unique indexing solution does not exist for mathematical reasons. For example, when a lattice metric singularity (LMS) occurs [3,4], there are two or more cells that will account for the same set of observed *d*-spacings.

Herein a quaternary lattice metric singularity and its impact on the characterization of Mawsonite and Chatkalite will be analyzed. In this case, there are four different lattices with three different symmetries (Table 1) that are consistent with the same set of *d*-spacings. This has caused considerable confusion and, over the years, Mawsonite has been reported in a variety of ways including pseudo cubic *I*, Tetragonal *I*, and Tetragonal *P*.

The research on these minerals demonstrates that in certain cases obtaining the unit cell is difficult. Caution must be taken to obtain the correct lattice. For example, when using indexing procedures, there is no inherent reason to assume the correct answer is necessarily the lattice with the highest symmetry. Clearly, in addition to indexing procedures, other methods — e.g., optical, single crystal, etc. — should be routinely employed to establish uniquely the lattice and symmetry.

## 2. Mawsonite and Chatkalite: A Quaternary Quandary

The minerals Mawsonite and Chatkalite serve to illustrate the role that lattice metric singularities play in the characterization of materials via powder indexing procedures. Over the years, Mawsonite (Table 2) has been characterized by a variety of unit cells. As shown in the Table, they include an *I*-centered cubic cell [7], an *I*-centered tetragonal cell [9] and a primitive tetragonal cell [10].

In the initial reporting of the mineral Mawsonite, Markham et al. [7] noted that the optical data was inconsistent with cubic symmetry so they used the phrase “body centred pseudocubic” in their description of the mineral. Yamanaka et al. [9], using the refinement program of Evans et al. [12], determined a tetragonal *I* cell with dimensions close to those of the initial cubic *I* cell. Single-crystal work by Szymański [10] established the crystal structure as tetragonal *P*. As Chatkalite (Fe^2+^Sn_2_ replaces Fe_2_^3+^Sn^4+^ of Mawsonite) is structurally very similar to Mawsonite, its powder pattern is indexed on the basis of a primitive tetragonal unit cell (*a* = 7.61(1) Å and *c* = 5.373(5) Å) [13]. In the latest Fleischer’s Glossary of Mineral Species [14], both Mawsonite and Chatkalite are listed as tetragonal.

There is an underlying mathematical reality that links and explains these diverse unit cells. The determinative *c*/*a* ratios for the tetragonal *P* cells for Mawsonite [10] and for Chatkalite [13] are almost equal to 1/√2. When this condition occurs, the experimentalist is confronted with a quaternary lattice metric singularity. In this case, therefore, there are four different lattices consistent with the same set of *d*-spacings. Thus from powder data alone one cannot uniquely assign the lattice. This is demonstrated in the next section.

## 3. The Quaternary Lattice Metric Singularity

The four lattices involved in the quaternary singularity are given in Table 1. The lattices I, II, III, and IV are defined by the *I*-centered cell 1, the primitive cell 2, the *F*-centered cell 3, and the primitive cell 4, respectively. When one compares the volumes of the reduced cells for lattices I, II, III, and IV, one notes that the cell volumes are in an 1:1/2:1/3:1/4 relationship. In fact, reduced cells 2, 3, and 4 are derivative subcells of cell 1.

The reduced forms for cells 1–4 that define the four lattices are also given in Table 1. As the reduced forms are all different, the four cells clearly define different lattices. The reduced forms [5] 5, 21, 26, and 32 are characteristic of an *I*-centered cubic, a primitive tetragonal, an *F*-centered orthorhombic and a primitive orthorhombic lattice, respectively. Detailed inspection of the 2nd, 3rd, and 4th reduced forms shows that there is more specialization than required for the given reduced form type [5]. For example, in the case of lattice II, the 1:2:2 relationship between the symmetrical dot products — ***a* · *a* : *b* · *b* : *c · c*** — of the reduced form implies a highly specialized lattice.

In Table 3, the unique *d*-spacings for the four lattices are given. The sets of unique interplanar spacings are identical. However, Table 3 shows that the number (*M*) of *d*-spacings with a given calculated *d*-value can be different for lattices I-IV. Consider a calculated *d*-value equal to 1.4615 Å. For this *d*-value, the Table shows that the numbers (*M*) calculated for lattices I, II, III, and IV are 1, 2, 9, and 3, respectively. For those cases in which the NBS*AIDS83 [15] program calculates more than one (not symmetrically related) *d*-spacing with the same value, the *hkl* indices for only the first of the group are given in the Table.

For the nonspecialized lattice of tetragonal or orthorhombic symmetry, the program would calculate *M* discrete *d*-spacings for those cases in which *M* > 1 in Table 3. Inspection of *d*-spacing data for lattices II, III, and IV reveals that these four lattices are highly specialized in the sense that the value of *M* is often greater than 1. Thus the patterns have far fewer discrete lines than normally possible for the given symmetry. This is shown in Table 4. In column 4 of this table, the compression ratio is given. This is the ratio of the unique *d*-spacings to the total calculated *d*-spacings. For the orthorhombic *F* lattice, the compression ratio is 0.359 when the *d*-spacings are calculated out to a 2θ of 110° with λ = 1.5405 Å.

### 3.1 Finding Potential Lattices

As Mawsonite and Chatkalite demonstrate, the researcher needs to be aware of all possible lattices consistent with a given set *d*-spacings. In the case of Cubic *I*, one knows (Table 3) that at least 3 additional cells fit the data equally well. Single crystal studies [10] showed the lattice for Mawsonite is tetragonal *P*.

In other cases, the existence of further possibilities may not be known. To determine such possibilities, the DICVOL [1] indexing program has proved to be a valuable tool. In a recent publication [16] on the history of the dichotomy method for powder indexing, Boultif points out that the strategies used in DICVOL04 are appropriate for dealing with the above type of mathematical ambiguities. In the DICVOL04 approach, the “symmetries are scanned separately from the highest symmetry (cubic) towards the lowest symmetry (triclinic), while searching first for the solutions with smaller volumes.” The case of Mawsonite and Chatkalite demonstrates that the correct solution is not necessarily the one with the higher crystal symmetry. Likewise for a given symmetry, the correct answer may not be the one with the smallest volume.

In any case, the researcher should always confirm the indexing solution with data from other techniques. This approach is the modus operandi for mineralogists. From the beginning Markham and Lawrence [7] were aware of a symmetry problem and used the phrase “body centered pseudo cubic” in the description of the new mineral. Even though they indexed Mawsonite on the basis of a Cubic *I* cell, they noted the mineral cannot be truly cubic because of its anisotropic optical properties. The correct lattice was determined a decade later by means of single crystal data [10].

Finally, it is prudent to analyze the reduced form of a cell obtained via indexing procedures. Is there more specialization than required by the given reduced form type? As a specialized reduced form is frequently associated with a high compression ratio, it serves as an indicator of the existence of additional indexing possibilities.

## 4. Conclusion

The tetragonal *P* lattice for the Mawsonite structure, as established by single crystal studies [10], is undoubtedly the correct lattice. As Chatkalite is presumed isostructural with Mawsonite, its powder data has been refined on the basis of a tetragonal unit cell [13]. In the 2004 version of Fleischer’s Glossary of Mineral Species [14], both Mawsonite and Chatkalite are listed with tetragonal symmetry.

The historical confusion on the symmetry of Mawsonite has a valid mathematical explanation. The lattices for Mawsonite and Chatkalite are unusual because they are highly specialized. In each case: (1) the *a*/*c* ratio is very near the square root of 2 (√2 = 1.4142, *a*/*c* for Chatkalite = 1.4163, *a*/*c* for Mawsonite = 1.4190);(2) the symmetrical scalars of the reduced form [***a* · *a* : *b* · *b* : *c* · *c*** = 1:2:2] have greater specialization than required for the given reduced form type; (3) the tetragonal lattice has derivative lattices of higher symmetry; and (4) the powder pattern is highly compressed (Table 4).

Mawsonite is an exemplar-type structure. It crystallizes in a lattice that corresponds to the second member in the quaternary lattice metric singularity and not to the first with highest symmetry (Table 1). This result has important implications in structure analysis. First, any cubic *I* lattice — established solely on the basis of indexing procedures — may actually be tetragonal or orthorhombic! Second, in determining the lattice of an unknown, results from powder data indexing procedures should routinely be confirmed by other techniques (e.g., single crystal, optical, etc.).

## Figures and Tables

**Table 1:** Quaternary lattice metric singularity. The four lattices yield the same set of unique calculated *d*-spacings. For each lattice, the table gives the conventional cell along with the corresponding reduced cell and reduced form^a^.

	Lattice I	Lattice II	Lattice III	Lattice IV
	Cubic *I*	Tetragonal *P*	Orthorhombic *F*	Orthorhombic *P*
	Conventional Cells			
Cell	Cell 1	Cell 2	Cell 3	Cell 4
*a*(Å)^b^	10.7400	7.5943	5.0629	3.7972
*b*(Å)	10.7400	7.5943	10.7400	5.3700
*c*(Å)	10.7400	5.3700	15.1887	7.5943
*α* (°)	90.0	90.0	90.0	90.0
*β* (°)	90.0	90.0	90.0	90.0
*γ* (°)	90.0	90.0	90.0	90.0
*V*(Å^3^)	1238.83	309.71	825.89	154.86
*a*/*c*	1.0	√2	1/3	1/2
*b*/*c*	1.0	√2	√½	√½
	Reduced Cells			
Cell	R1	R2^c^	R3^d^	R4^e^
*a*(Å)	9.3011	5.3700	5.0629	3.7972
*b*(Å)	9.3011	7.5943	5.9368	5.3700
*c*(Å)	9.3011	7.5943	8.0051	7.5943
*α* (°)	109.471	90.0	82.251	90.0
*β* (°)	109.471	90.0	71.565	90.0
*γ* (°)	109.471	90.0	64.761	90.0
*V*(Å^3^)	619.42	309.71	206.47	154.86
	Reduced Forms			
Form	RF1	RF2	RF3	RF4
***a* · *a***(Å^2^)	86.511	28.837	25.633	14.419
***b* · *b***(Å^2^)	86.511	57.673	35.245	28.837
***c* · *c***(Å^2^)	86.511	57.673	64.082	57.673
***b* · *c***(Å^2^)	–28.837	0	6.408	0
***a* · *c***(Å^2^)	–28.837	0	12.816	0
***a* · *b***(Å^2^)	–28.837	0	12.816	0
	Normalized Reduced Forms			
Form	F1	F2	F3	F4
***a* · *a***	1	1	1	1
***b* · *b***	1	2	1.375	2
***c* · *c***	1	2	2.500	4
***b* · *c***	− ⅓	0	¼	0
***a* · *c***	− ⅓	0	½	0
***a* · *b***	− ⅓	0	½	0
Reduced Form# [5]	5	21	26	32

a NIST*LATTICE [6] was used to determine lattice relationships given in the table and text.

b Note: 1 Å [= 0.1nm = 10^−10^m] is the common unit in crystallography.

Transformations

c R2 → R1 [1 −1 0 / −1 0 1 / −1 0 −1] Δ = 2

d R3 → R1 [1 1 0 / −2 1 0 / 0 −1 1] Δ = 3

e R4 → R1 [0 −1 −1 / 2 1 0 / 0 −1 1] Δ = 4

(where Δ = the determinate of the given matrix)

**Table 2:** Comparison of reported diffraction data on Mawsonite

	*d*-spacings^a^ Cubic *I*				Case A^b^ Markham et al. [7]		Case B^c^ Ottemann et al. [8]		Case C^d^ Yamanaka et al. [9]		Case D^e^ Szymański [10]		Case E^f^ Kissin et al. [11]	
No	*hkl*			*d*-calc	*d*-obs	*I*	*d*-obs	*I*	*d*-obs	*I*	*d*-calc	*I*	*d*-obs	*I*
1	1	1	0	7.5943					7.62	3				
2	2	0	0	5.3700	5.37	20			5.38	10	5.37	11	5.3	1
3	2	1	1	4.3846	4.37	20			4.38	15	4.38	14	4.38	4
4	2	2	0	3.7972	3.80	10			3.80	8	3.800	6	3.70	½
5	3	1	0	3.3963	3.34	10			3.378	4				
6	2	2	2	3.1004	3.09	100	3.105	m	3.099	100	3.100	100	3.10	10
7	3	2	1	2.8704	2.875	20			2.868	10	2.871	13	2.88	4
8	4	0	0	2.6850	2.680	50	2.652	w	2.684	25	2.683	26	2.69	6
9	3	3	0	2.5314					2.462	3			2.52	5
10	4	2	0	2.4015	2.395	10			2.401	8	2.401	6	2.41	3
11	3	3	2	2.2898	2.287	10			2.291	5	2.291	4	2.30	2
12	4	2	2	2.1923	2.185	5			2.192	3	2.190	2	2.19	1
13	5	1	0	2.1063	2.098	5							2.09	½
14	5	2	1	1.9608	1.959	5			1.962	3	1.962	3	1.958	½
15	4	4	0	1.8986	1.895	80	1.887	s	1.899	75	1.899	70	1.899	7
16	5	3	0	1.8419										
17	6	0	0	1.7900	1.788	5			1.791	3	1.790	3	1.790	2
18	6	1	1	1.7423	1.739	5			1.742	3	1.742	3	1.741	2
19	6	2	0	1.6981										
20	5	4	1	1.6572										
21	6	2	2	1.6191	1.618	60	1.612	w	1.620	40	1.619	43	1.618	6
22	6	3	1	1.5835							1.581	2	1.584	½
23	4	4	4	1.5502	1.547	10			1.549	7	1.550	6	1.552	3
24	5	5	0	1.5189										
25	6	4	0	1.4894										
26	7	2	1	1.4615	1.460	5			1.463	3	1.462	3	1.462	2
27	6	4	2	1.4352									1.438	½
28	7	3	0	1.4102										
29	6	5	1	1.3640					1.366	3	1.365	3	1.362	½
30	8	0	0	1.3425	1.343	20	1.333	w	1.344	15	1.341	13	1.338	5
31	7	4	1	1.3220					1.318	3				
32	8	2	0	1.3024					1.302	4	1.303	1		
33	6	5	3	1.2837										
34	6	6	0	1.2657										
35	7	5	0	1.2485										
36	6	6	2	1.2320	1.232	30	1.228	m	1.233	10	1.232	20		
37	7	5	2	1.2161									1.213	½
38	8	4	0	1.2008	1.201	5	1.199	w	1.201	5b	1.201	9b	1.201	3
39	9	1	0	1.1860										
40	8	4	2	1.1718										
41	9	2	1	1.1581									1.155	½
–	–			–										
45	8	4	4	1.0961		50	1.093	m	1.096	15	1.096	34	1.095	7
46	7	7	0	1.0849										
47	8	6	0	1.0740										
48	10	1	1	1.0634	1.065	50								
49	10	2	0	1.0531										
50	9	5	0	1.0432										
51	10	2	2	1.0335	1.034	20	1.031	w			1.033	22	1.032	5
52	10	3	1	1.0240									1.023	½
53	8	7	1	1.0059										
54	10	4	0	0.9972									0.9967	½
–	–			–										
58	10	5	1	0.9568									0.9567	½
59	8	8	0	0.9493	0.950	20					0.949	25	0.9491	5
–	–			–										

a Calculated *d*-spacings (Cubic *I*: *a* = 10.7400 Å, *V* = 1238.83 Å^3^).

b Cubic *I* with *a* =10.74(1) Å (Mawsonite observed powder data indexed).

c Cubic *I* with *a* =10.710(5) Å (Germanium Mawsonite observed powder data indexed).

d Tetragonal *I* with *a* = 10.745(1) Å and *c* = 10.711(6) Å (Mawsonite observed powder data indexed).

e Tetragonal *P* with *a* = 7.603(2) Å and *c* = 5.358(1) Å (Cell and structure determined by single-crystal x-ray analysis. Powder pattern calculated).

F Tetragonal *P* with *a* = 7.603(2) Å and *c* = 5.358(1) Å (Mawsonite observed powder pattern indexed).

**Table 3:** Quaternary lattice metric singularity. For the four lattices, the values of the calculated *d*-spacings (Å) are identical

	Lattice I: Cubic *I*^a^					Lattice II: Tetragonal *P*^b^					Lattice III: Orthorhombic *F*^c^					Lattice IV: Orthorhombic *P*^d^				
No	*hkl*			*d*-calc	*M*^e^	*hk*l			*d*-calc	*M*	*hkl*			*d*-calc	*M*	*hkl*			*d*-calc	*M*
1	1	1	0	7.5943	1	1	0	0	7.5943	1	0	0	2	7.5943	1	0	0	1	7.5943	1
2	2	0	0	5.3700	1	1	1	0	5.3700	2	0	2	0	5.3700	1	0	1	0	5.3700	1
3	2	1	1	4.3846	1	1	0	1	4.3846	1	0	2	2	4.3846	2	0	1	1	4.3846	1
4	2	2	0	3.7972	1	2	0	0	3.7972	2	0	0	4	3.7972	1	0	0	2	3.7972	2
5	3	1	0	3.3963	1	2	1	0	3.3963	1	1	1	3	3.3963	1	1	0	1	3.3963	1
6	2	2	2	3.1004	1	2	0	1	3.1004	1	0	2	4	3.1004	1	0	1	2	3.1004	2
7	3	2	1	2.8704	1	2	1	1	2.8704	1	1	3	1	2.8704	1	1	1	1	2.8704	1
8	4	0	0	2.6850	1	2	2	0	2.6850	2	0	4	0	2.6850	1	0	2	0	2.6850	2
9	3	3	0	2.5314	1	3	0	0	2.5314	2	0	4	2	2.5314	5	0	0	3	2.5314	2
10	4	2	0	2.4015	1	3	1	0	2.4015	3	2	0	2	2.4015	1	1	1	2	2.4015	1
11	3	3	2	2.2898	1	3	0	1	2.2898	1	0	2	6	2.2898	2	0	1	3	2.2898	1
12	4	2	2	2.1923	1	3	1	1	2.1923	2	0	4	4	2.1923	2	0	2	2	2.1923	2
13	5	1	0	2.1063	1	3	2	0	2.1063	2	1	3	5	2.1063	2	1	0	3	2.1063	2
14	5	2	1	1.9608	1	3	2	1	1.9608	1	1	5	1	1.9608	3	1	1	3	1.9608	1
15	4	4	0	1.8986	1	4	0	0	1.8986	2	0	0	8	1.8986	1	0	0	4	1.8986	3
16	5	3	0	1.8419	1	4	1	0	1.8419	2	1	5	3	1.8419	3	0	2	3	1.8419	2
17	6	0	0	1.7900	1	3	3	0	1.7900	4	0	6	0	1.7900	4	0	1	4	1.7900	3
18	6	1	1	1.7423	1	4	1	1	1.7423	2	0	6	2	1.7423	2	0	3	1	1.7423	2
19	6	2	0	1.6981	1	4	2	0	1.6981	3	2	2	6	1.6981	1	1	0	4	1.6981	2
20	5	4	1	1.6572	1	3	2	2	1.6572	1	1	5	5	1.6572	3	1	2	3	1.6572	1
21	6	2	2	1.6191	1	4	2	1	1.6191	2	0	6	4	1.6191	1	0	3	2	1.6191	4
22	6	3	1	1.5835	1	2	1	3	1.5835	1	1	1	9	1.5835	2	1	3	1	1.5835	1
23	4	4	4	1.5502	1	4	0	2	1.5502	1	0	4	8	1.5502	1	0	2	4	1.5502	2
24	5	5	0	1.5189	1	5	0	0	1.5189	2	0	0	10	1.5189	3	0	0	5	1.5189	3
25	6	4	0	1.4894	1	5	1	0	1.4894	3	2	4	6	1.4894	1	1	3	2	1.4894	1
26	7	2	1	1.4615	1	4	3	1	1.4615	2	1	7	1	1.4615	9	0	1	5	1.4615	3
27	6	4	2	1.4352	1	5	1	1	1.4352	3	2	6	2	1.4352	1	1	2	4	1.4352	2
28	7	3	0	1.4102	1	5	2	0	1.4102	1	1	7	3	1.4102	1	1	0	5	1.4102	1
29	6	5	1	1.3640	1	5	2	1	1.3640	2	2	6	4	1.3640	2	1	1	5	1.3640	2
30	8	0	0	1.3425	1	4	4	0	1.3425	2	0	8	0	1.3425	1	0	4	0	1.3425	2
31	7	4	1	1.3220	1	4	3	2	1.3220	2	0	8	2	1.3220	7	0	2	5	1.3220	3
32	8	2	0	1.3024	1	5	3	0	1.3024	5	0	6	8	1.3024	2	0	3	4	1.3024	3
33	6	5	3	1.2837	1	4	1	3	1.2837	1	1	5	9	1.2837	2	2	3	1	1.2837	1
34	6	6	0	1.2657	1	6	0	0	1.2657	4	0	8	4	1.2657	5	0	0	6	1.2657	4
35	7	5	0	1.2485	1	6	1	0	1.2485	3	1	3	11	1.2485	3	1	2	5	1.2485	3
36	6	6	2	1.2320	1	6	0	1	1.2320	2	0	2	12	1.2320	2	0	1	6	1.2320	4
37	7	5	2	1.2161	1	6	1	1	1.2161	1	1	7	7	1.2161	3	3	1	1	1.2161	1
38	8	4	0	1.2008	1	6	2	0	1.2008	3	4	0	4	1.2008	1	1	0	6	1.2008	4
39	9	1	0	1.1860	1	5	4	0	1.1860	2	0	8	6	1.1860	3	0	4	3	1.1860	2
40	8	4	2	1.1718	1	6	2	1	1.1718	3	2	8	2	1.1718	3	1	1	6	1.1718	2

a Cell 1 (Cubic *I*): *a* = 10.7400 Å, *V* = 1238.83 Å^3^.

b Cell 2 (Tetragonal *P*): *a* = 7.5943 Å, *c* = 5.3700 Å, *V* = 309.71 Å^3^.

c Cell 3 (Orthorhombic *F*): *a* = 5.0629 Å, *b* = 10.7400 Å, *c* = 15.1887 Å, *V* = 825.89 Å^3^.

d Cell 4 (Orthorhombic *P*): *a* = 3.7972 Å, *b* = 5.3700 Å, *c* = 7.5943 Å, *V* = 154.86 Å^3^.

e Number of lines calculated (NBS*AIDS83 [15]) with the specified *d*-spacing value.

**Table 4:** Quaternary lattice metric singularity. The *d*-spacings for each lattice were calculated^a^ using the specified 2θ maximum values and λ = 1.5405 Å. The number of unique *d*-spacings for the three lattices is identical. The low values for the compression ratios for lattices II, III, & IV show that they are specialized (i.e. many *d*-spacings have the same value).

	2θ Maximum	Unique *d*-spacings	Total *d*-spacings	Compression Ratio^b^
Cell 1^c^ Lattice I	80	38	38	1
	90	45	45	1
	100	53	53	1
	110	60	60	1
	120	67	67	1
Cell 2^d^ Lattice II	80	38	76	0.500
	90	45	92	0.489
	100	53	116	0.457
	110	60	135	0.444
	120	67	156	0.429
Cell 3^e^ Lattice III	80	38	85	0.447
	90	45	112	0.402
	100	53	140	0.379
	110	60	167	0.359
	120	67	191	0.351
Cell 3^f^ Lattice IV	80	38	77	0.494
	90	45	95	0.474
	100	53	120	0.442
	110	60	138	0.435
	120	67	165	0.406

a NBS*AIDS83 [15].

b Compression ratio = “unique *d*-spacings/possible *d*-spacings” for a given symmetry.

c Cell 1 (Cubic *I*): *a* = 10.7400 Å, *V* = 1238.83 Å^3^.

d Cell 2 (Tetragonal *P*): *a* = 7.5943 Å, *c* = 5.3700 Å, *V* = 309.71 Å^3^.

e Cell 3 (Orthorhombic *F*): *a* = 5.0629 Å, *b* = 10.7400 Å, *c* = 15.1887 Å, *V* = 825.89 Å^3^.

F Cell 3 (Orthorhombic *P*): *a* = 3.7972 Å, *b* = 5.3700 Å, *c* = 7.5943 Å, *V* = 154.86 Å^3^.
